# Role of PPE18 Protein in Intracellular Survival and Pathogenicity of *Mycobacterium tuberculosis* in Mice

**DOI:** 10.1371/journal.pone.0052601

**Published:** 2012-12-28

**Authors:** Khalid Hussain Bhat, Asma Ahmed, Santosh Kumar, Pawan Sharma, Sangita Mukhopadhyay

**Affiliations:** 1 Centre for DNA Fingerprinting and Diagnostics (CDFD), Nampally, Hyderabad, India; 2 International Centre for Genetic Engineering & Biotechnology, ICGEB Campus, Aruna Asaf Ali Marg. New Delhi, India; 3 North Eastern Region-Biotechnology Programme Management Cell (NER-BPMC), A-254, Defence Colony, Bhisham Pitamah Marg, New Delhi, India; University of Hyderabad, India

## Abstract

**Background:**

Ever since its discovery the mycobacterial proline-proline-glutamic acid (PPE) family of proteins has generated a huge amount of interest. Understanding the role of these proteins in the pathogenesis of *Mycobacterium tuberculosis* (Mtb) is important. We have demonstrated earlier that the PPE18 protein of Mtb induces IL-10 production in macrophages with subsequent downregulation of pro-inflammatory cytokines like IL-12 and TNF-α and favors a T-helper (Th) 2-type of immune response.

**Methodology/Principal Findings:**

Using a *ppe18* genetic knock-out Mtb strain, we have now carried out infection studies in mice to understand the role of PPE18 in Mtb virulence. The studies reveal that lack of PPE18 leads to attenuation of Mtb *in vivo*. Mice infected with the *ppe18* deleted strain have reduced infection burden in lung, liver and spleen and have better survival rates compared to mice infected with the wild-type Mtb strain.

**Conclusions/Significance:**

Taken together our data suggest that PPE18 could be a crucial virulence factor for intracellular survival of Mtb.

## Introduction

Tuberculosis (TB), both pulmonary and extrapulmonary, is a major global health concern. According to the World Health Organization, 1.3 million people died of tuberculosis in 2008 and 34% of the victims were from South-East Asia. Also, one third of the world population is infected with Mtb asymptomatically. TB also accounts for 32% of deaths among AIDS afflicted individuals [1]. Mtb is a highly successful pathogen. It has developed several efficient strategies to survive and replicate in the macrophage, its primary host cell [2]. These strategies include prevention of fusion of phagosome and lysosome, deterrence of phagosome acidification, expression of virulence proteins, protection from reactive oxygen species (ROS), inhibition of protective cytokines like interleukin (IL)-12, tumor necrosis factor (TNF)-α, evasion of antigen presentation [3]–[6] and inhibition of apoptosis [7]. Mtb turns down the T helper (Th) 1-type immune response which is beneficial to the host and up-regulates Th2-type cytokines which are anti-inflammatory and helpful for its survival [8]. The bacilli with the help of these mechanisms live inside a human host, sometimes for years together. Some of these mechanisms are well understood while others remain to be comprehended. Understanding the host-pathogen interactions during Mtb infection will help immensely in combating the menace of tuberculosis worldwide.

The acid rich proline-glutamic acid (PE)/PPE family of proteins is exclusive to mycobacteria. Unraveling of the Mtb genome revealed that 10% of its coding ability is devoted to the PE and PPE families, comprising of 99 and 68 members respectively [9], [10]. There is a gradual expansion of PE/PPE proteins from non-pathogenic to pathogenic mycobacteria [11]. PE and PPE family members are characterized by the presence of conserved proline-glutamic acid or proline-proline-glutamic acid motifs of 110 and 180 amino acids respectively at the N-terminal region [9], [12]. The C-terminal region is found to be highly variable [13]. Depending upon the presence of characteristic repeats, the PE and PPE families can be divided into subfamilies [11]. The PE_PGRS and PPE_MPTR subfamilies have long stretches of GC rich repeats which are believed to be the hotspots for recombination events and other mutations. This leads to a great deal of sequence variation and polymorphism in these proteins [14]. There is also speculation that this high variability may contribute to the antigenic variation that helps the pathogen to evade host protective immune responses [15]. Not much information is available about the exact patho-physiological role of PE/PPE proteins. However, there is evidence to suggest that they are up-regulated during stress conditions and may hence facilitate bacterial survival during infection [16], [17]. Also, it has been found that PE/PPE proteins modulate macrophage protective functions, cytokine secretion, apoptosis and necrosis of host cells [16], [18]–[20].

PPE18 (Rv1196), also known as Mtb39a, a member of the PPE family, is expressed more in Mtb as compared to *Mycobacterium bovis* [21]. Previous work by us documents that PPE18 binds to the toll like receptor (TLR) 2 on macrophages, and *via* activation of the p38 mitogen activated protein kinase (MAPK) pathway, it up-regulates IL-10 cytokine production. Also, its interaction with TLR2 leads to phosphorylation of the suppressor of cytokine signaling (SOCS) 3 protein which then physically interacts with the IκBα-nuclear factor (NF)-κB/c-rel complex. This interaction prevents the nuclear translocation of p50 and p65 NF-κB and c-rel transcription factors. As a consequence, there is a downregulation of transcription of NF-κB-regulated genes like IL-12 and TNF-α. Thus, PPE18 was found to selectively downregulate the proinflammatory and Th1-type immune response [5], [22]. At the same time, it increases secretion of IL-10 [22] which favors a Th2-type response. Also, our previous work reveals that macrophages infected with Mtb strain lacking PPE18 produce less IL-10 and more IL-12 p40 compared to those infected with wild-type Mtb strain. Interestingly, PPE18 was shown to skew the anti-PPD Th1 response towards the Th2-type in T cells isolated from PBMCs from BCG vaccinated individuals [22]. To understand the role of PPE18 in Mtb virulence *in vivo*, in the present study, the *ppe18* knock-out (KO) strain was used in a murine model of Mtb infection. Our studies revealed that mice infected with *ppe18* KO bacteria had lower bacterial load, less tissue pathology and improved survival rates compared to mice infected with wild-type (WT) bacteria. These data reveal that PPE18 probably plays an important role in the survival and multiplication of Mtb bacilli during infection.

## Materials and Methods

### Bacterial Strains

The Mtb *ppe18* knock-out (KO) strain (*ppe18* null mutant; mutant ID 1440, MT1234) and its corresponding wild-type (WT) strain (CDC1551) were obtained from Colorado State University (as part of National Institutes of Health National Institutes of Health NIAID Contract No. HHSN266200400091C entitled, “Tuberculosis Vaccine Testing and Research Materials”). The *M. tuberculosis* strains were grown in Difco Middlebrook 7H9 broth (BD Biosciences, Sparks, MD, USA) supplemented with 0.2% glycerol (Sigma-Aldrich, St. Louis, MO), 0.5% Tween 80 (Sigma-Aldrich), and 10% oleic acid albumin dextrose complex (OADC, BD Biosciences) in a biosafety level 3 (BSL-3) facility at International Centre for Genetic Engineering and Biotechnology (ICGEB), New Delhi, India.

### Mouse Infections

Four to six weeks old C57Bl/6 mice of either sex were kept in individually ventilated cages in a BSL-3 animal house at ICGEB, New Delhi. Animal experiments were conducted at the animal house facility of ICGEB, New Delhi, India according to the guidelines of the Institutional Animal Ethics Committee. Mice were infected with 1×10^8^ of WT or *ppe18* KO Mtb strain by aerosol route using the Madison Aerosol Chamber (University of Wisconsin, Madison, WI) pre-calibrated to deliver small inocula of bacilli (delivering about 80–130 bacilli of both the strains per lung of animal sacrificed at day one as assessed by killing two mice 24 hours after exposure to aerosol and plating the lungs homogenates on nutrient 7H11 agar and counting CFU after a 21 days incubation at 37°C).

### Infection Burden in Organs

Bacterial loads in lung, liver, and spleen were evaluated at different time points after aerosol infection with WT or *ppe18* KO Mtb to follow the course of infection. For this, lung, liver and spleen were aseptically removed from euthanized animals from each group. Organs were homogenized in sterile saline containing 0.05% Tween 80 (Sigma-Aldrich). Serial dilutions of homogenized organs were plated on 7H11 plates supplemented with 10% OADC (BD Biosciences). Plates were incubated at 37°C and colonies were counted after 21 days.

### Histopathology

Lung, liver and spleen of mice infected by aerosol route with either WT or *ppe18* KO Mtb strain were aseptically removed from euthanized animals and were fixed in 10% formalin and then embedded in paraffin wax. Sections were then stained with hematoxylin and eosin (H&E) stain for visualizing mammalian cells. Sections were visualized under an Olympus CX21 microscope (Olympus, Japan). Also, microphotographs were taken using an Olympus DP72 CCD camera attached to the microscope. DP2-BSW software was used for image analysis.

### Statistical Analysis

Data analysis was performed using the Student’s *t* test, considering *P* values <0.05 to be significant. Values are presented as mean bacterial count ± standard error of the mean (SEM) of 5 animals per group per time point [23], [24].

## Results

### PPE18 Confers a Growth Advantage to Mtb *in vivo* in a Mouse Model of Infection

**Figure 1:**
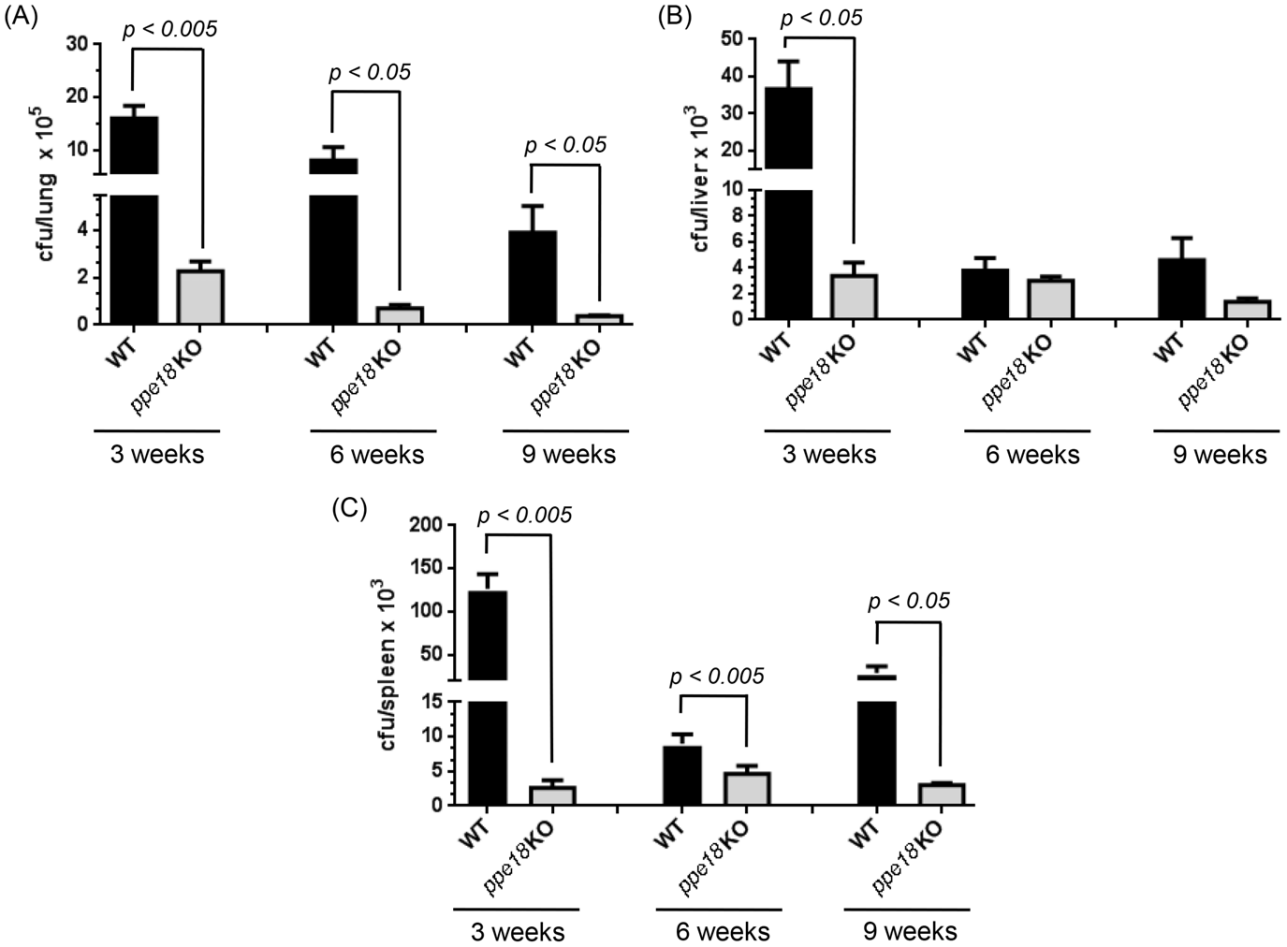
Infection burden in mice infected with wild-type (WT) or *ppe18* knock-out (KO) Mtb strain. C57BL/6 mice were infected aerogenically with a low dose (1×10^8^) of either WT or *ppe18* KO strains of Mtb. At different time points post infection, mice were sacrificed and CFU counts were measured in lung (A), liver (B) and spleen (C). Data are mean ± SEM of results for five mice per group for each time point.

To assess the role of PPE18 in growth of Mtb *in vivo*, C57Bl/6 mice were infected with either WT or *ppe18* KO strains of Mtb *via* the aerosol route and the bacterial burden was estimated in lung, liver and spleen of infected animals at 3 different time points (3 weeks, 6 weeks and 9 weeks) after infection. The aerosol infection deposited 80–130 colony forming units (CFUs) per lung (as assessed by counting CFUs in two infected mice per Mtb strain at day 1 post infection) Infection through aerosol deposits Mtb directly into the lungs and hence considered to be closest to physiological mode of infection. Lungs being the primary site of infection showed maximum CFUs at all the time points examined (Figure 1). In mouse model upon infection, bacteria are known to disseminate from lungs to liver and spleen [25]. It has been shown that in mice infected with a low dose of Mtb by aerosol route, bacterial numbers increase steadily with time to reach a peak at about 3 weeks and thereafter may decrease considerably [26], [27] when the host cell-mediated immune responses are high [25], [28]–[30]. Similarly, we also observed a steady rise in the bacterial load in all the organs till 3 weeks after aerosol infection and then a decrease at 6 weeks and 9 weeks post infection. Interestingly, we found that the number of *ppe18* KO bacteria remained significantly less in all the organs at almost all the time points investigated (Figure 1). In the lungs of *ppe18* KO-infected mice, the mean bacterial counts (± SEM) were significantly lower at 3 weeks post infection as compared to those of infected with WT Mtb strain and this trend continued to later time points also (6 weeks and 9 weeks) (Figure 1A). Similar observations were made in liver (Figure 1B) as well as in spleen (Figure 1C). These data indicate that the *ppe18* KO strain probably failed to multiply as robustly as the wild-type strain during the acute phase of infection *in vivo*. PPE18 has previously been reported to be non-essential for bacterial growth *in vitro* [31]. Our results indicate that PPE18 probably plays a role in replication and survival of Mtb *in vivo* and therefore, may be a candidate virulent factor.

### Mice Infected with *ppe18* KO Strain Show a Reduced Degree of Inflammation and Tissue Damage as Compared to mice Infected with WT Mtb Strain

**Figure 2:**
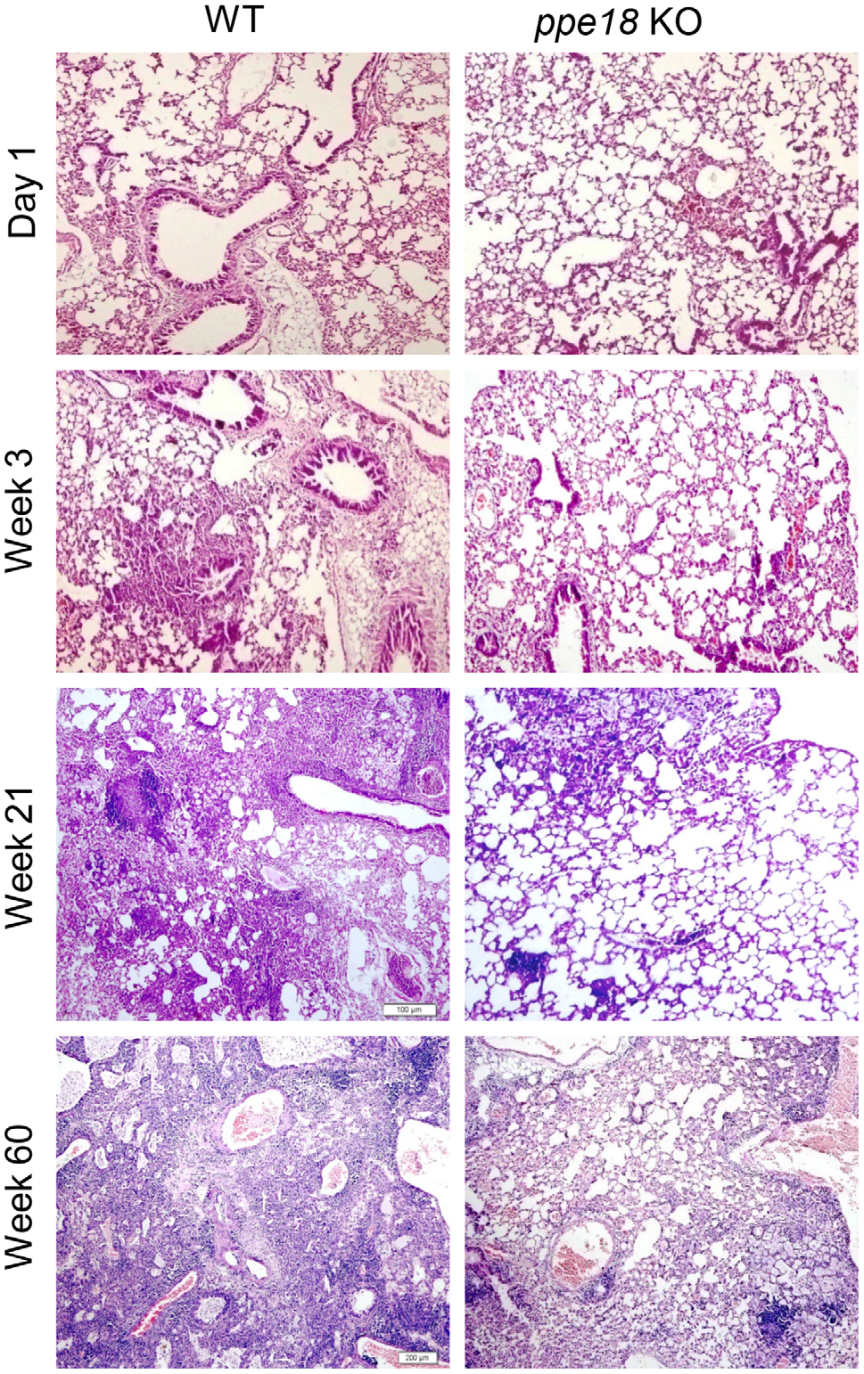
Histopathology of lungs from mice infected with wild-type (WT) or *ppe18* knock-out (KO) Mtb strain. Lung sections from mice following infection with either WT (left panel) or *ppe18* KO (right panel) strains of Mtb were stained with hematoxylin and eosin (H&E) stain at different time points post infection. Photographs of representative sections from 2 mice visualized at 40X magnification are shown.

**Figure 3:**
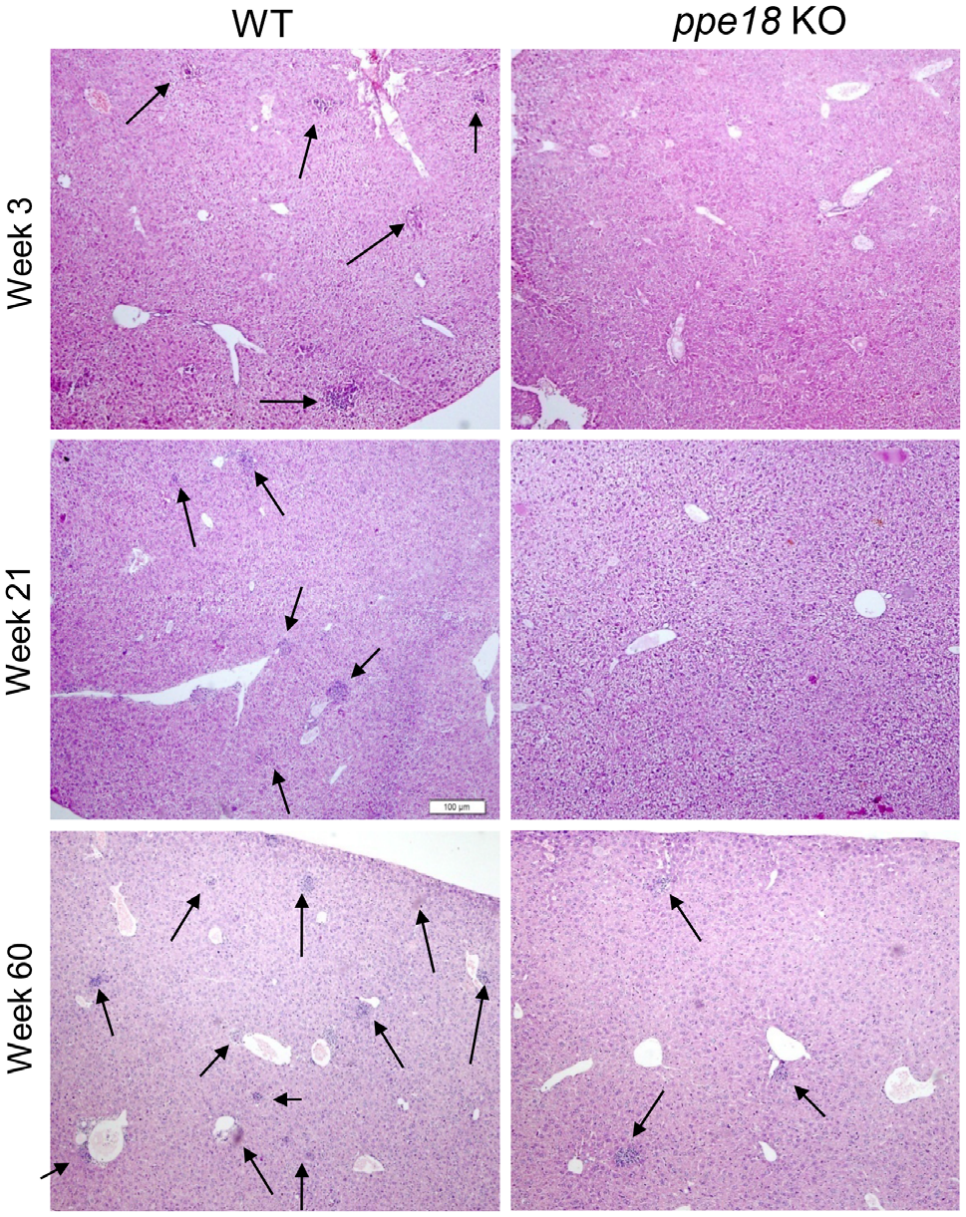
Histopathology of livers from mice infected with wild-type (WT) or Liver sections from mice infected with either WT (left panel) or *ppe18* KO (right panel) strains of Mtb were stained with hematoxylin and eosin (H&E) at different time points post infection. Photographs of representative sections from 2 mice visualized at 40X magnification are shown. Arrows indicate the sites of lymphocytic infiltration.

**Figure 4:**
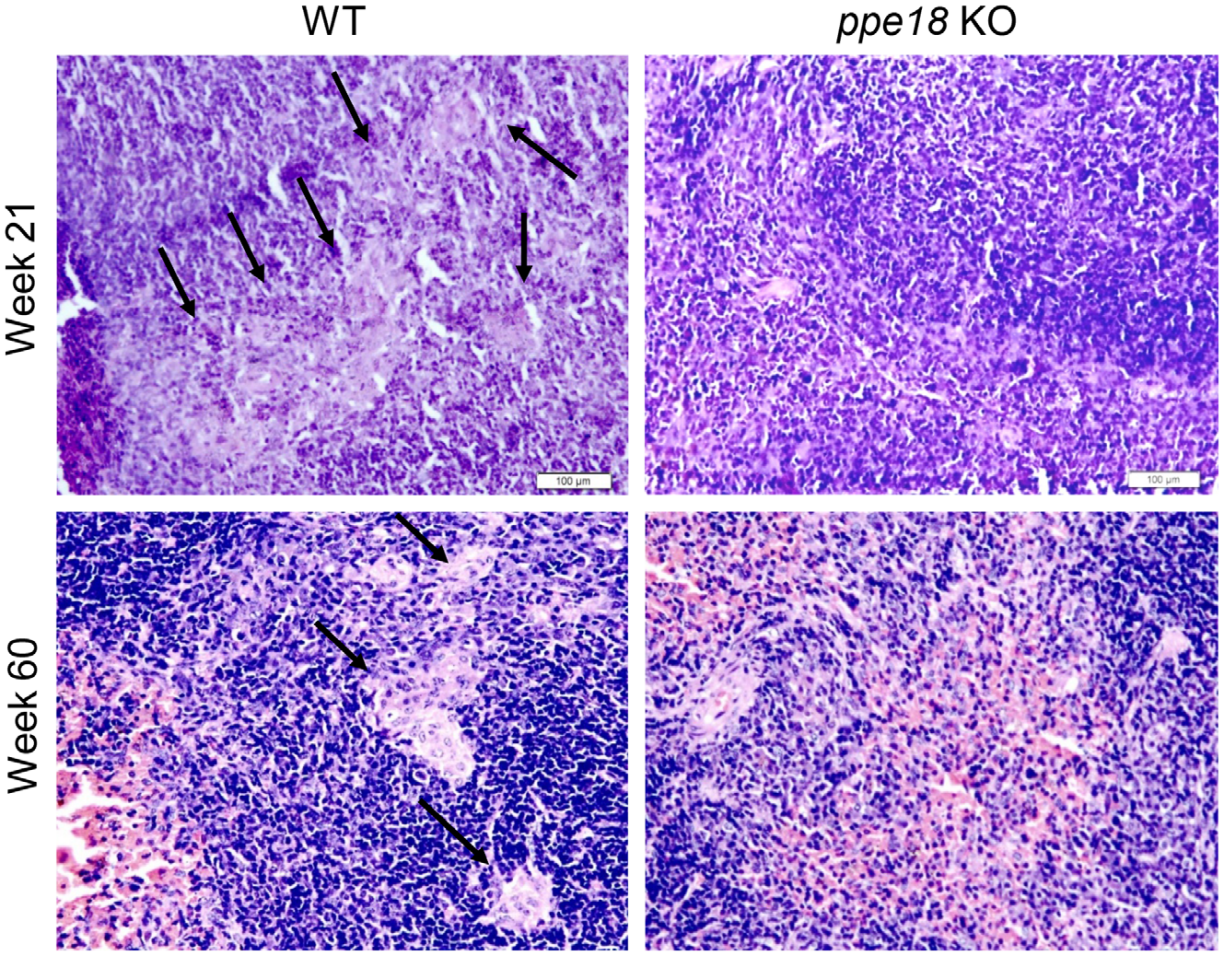
Histopathology of spleens from mice infected with wild-type (WT) or *ppe18* knock-out (KO) Mtb strain. Spleen sections from mice infected with either WT (left panel) or *ppe18* KO (right panel) strains of Mtb were stained with hematoxylin and eosin (H&E) at different time points post infection. Photographs of representative sections visualized at 200X magnification are shown. Arrows indicate the sites of macrophage infiltration.

Aerosol infection deposits Mtb directly in the lungs where their numbers slowly increase reaching a peak at about 3 weeks post infection. This is also the time when the influx of immune cells into the lungs is very high [28]–[30]. From the lungs the infection disseminates to liver and spleen. It has been reported that upon initiation of infection, bacteria in the lungs are lodged in the alveolar macrophages, myeloid dendritic cells (DCs) and neutrophils which form the first line of defense [25]. T cells first get activated in the draining lymph node and then migrate to the lungs about 14–21 days post infection [32]. In mice, the immune cells (macrophages and lymphocytes) are not arranged in the form of well defined granulomas that are observed in humans. It has also been observed that the immune response in the mouse model is often exaggerated and contributes to the aggravated tissue pathology resulting in death of the host even though the bacterial numbers are not significantly high [25]. Therefore, we examined the tissue damage in lung, liver and spleen in mice infected with WT and *ppe18* KO Mtb strains *in vivo* by histopathological analyses. The extent of inflammation and tissue damage due to infection as seen in the hematoxylin and eosin (H&E) stained sections of lung and liver from mice infected with WT Mtb was found to be markedly pronounced (Figures 2 and 3, left panel) than that observed in mice infected with the *ppe18* KO Mtb (Figures 2 and 3, right panel). The lungs of animals infected with WT or *ppe18* KO strains of Mtb became infiltrated with lymphocytes and macrophages at later time points after aerosol infection. However, the infiltration and lesions were more severe in mice infected with WT Mtb. Mice infected with *ppe18* KO had more intact alveolar spaces while mice infected with WT Mtb almost had none, especially at 21 and 60 weeks post infection (Figure 2). The lesions and tissue damage observed in the WT Mtb-infected animals were graded 4 (marked with 51–75% tissue affected) and 5 (severe with 76–100% tissue affected) and that in the *ppe18* KO-infected animals were graded 3 (moderate with 26–50% tissue affected), 60 weeks post infection (Figure 2). A similar trend was observed in the liver. The lesions observed in liver were, however, less severe compared to those in the lungs. Small foci of lymphocytic infiltration began to appear 3 weeks post infection in WT-infected mice (Figure 3). The foci were better observed at a magnification of 100X (Figure S1). The lesions became more numerous by 60 weeks post infection (Figure 3). Liver sections from *ppe18* KO-infected mice seemed normal at 3 and 21 weeks post infection and this correlated well with low CFU counts in the liver (Figure 1B), mild lymphocytic infiltration was observed in livers of *ppe18* KO-infected mice only at 60 weeks post infection (Figure 3). Effect of infection was not observed in spleen of both WT- and *ppe18* KO Mtb infected mice sacrificed at 3 weeks. Histiocytosis or accumulation of macrophages in spleen was observed at 21 and 60 weeks post infection in mice infected with WT Mtb strain (Figure 4), however, the spleen tissue structure of *ppe18* KO strain-infected mice appeared to be normal (Figure 4). Our observations from the histological slides indicated that in comparison to the WT, the *ppe18* KO strain elicited a reduced and delayed inflammatory response in lung, liver and spleen of the infected mice.

### Infection with *ppe18* KO Strain of Mtb Exhibits Reduced Tuberculosis Induced Fatality

**Figure 5:**
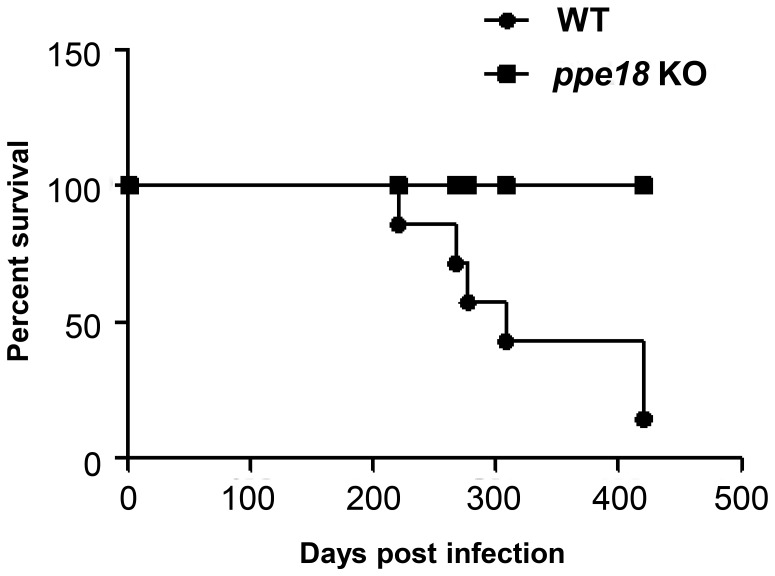
Survival of mice infected with wild-type (WT) or *ppe18* knock-out (KO) Mtb strain. Survival of C57BL/6 mice following a low-dose aerosol infection with either WT or *ppe18* KO strains of Mtb was monitored for 420 days post infection. The starting number of mice in each group was 8.

To reckon the total effect of *in vivo* growth and inflammation, survival of mice infected with WT and *ppe18* KO strains of Mtb was monitored over a prolonged period of time. No deaths were registered in the group of mice infected with the *ppe18* KO strain during the entire study period of 60 weeks. However, in the group of mice infected with the WT Mtb, survival rate had dropped to 25% 60 weeks post infection (Figure 5). Also, mice infected with *ppe18* KO strain visibly appeared healthier. The percentage increase in the weight of mice infected with *ppe18* KO strain 9 weeks after infection was 55±2% compared to the 31.9±5% increase in the weight of mice infected with the WT strain (Figure S2).

## Discussion

The virulence of Mtb is complicated and multifaceted. Earlier *in vitro* studies by us had indicated that PPE18 might aid in virulence of Mtb by favoring the non-protective anti-inflammatory Th2-type response and downregulating the protective pro-inflammatory and/or Th1-type immune response [5], [22]. The present infection studies in a mouse model have indeed revealed that deletion of PPE18 reduces the capacity of Mtb to multiply *in vivo*. Both *in vitro* [5], [22] and *in vivo* studies thus point to the fact that PPE18 probably plays an important role in the virulence of Mtb.

It is documented that immunity to Mtb in host depends on robust Th1-type T cell response while Th2-type response leads to enhanced susceptibility to mycobacterial infection [33]. Among the several factors that regulate T cell polarization and Th1/Th2 development, the cytokines produced by the activated macrophages have the most influential role [34], [35]. IL-12 [36]–[39] and TNF-α [40], [41] cytokines are known to trigger the anti-mycobacterial protective Th1-type immune response [38], [42], [43]. In contrast, IL-10 cytokine not only perturbs the Th1 response but also polarizes the T-cell immune response towards the non-protective Th2-type and promotes Mtb survival inside the host [44]–[48]. IL-10 has been shown to be linked with the ability of Mtb to evade immune responses and mediate long-term infections in the lungs [49]. Thus, mycobacterial strategy at every step is likely to perturb the macrophage IL-12/IL-10 balance to subsequently establish a Th2-type response. This hypothesis is supported by the observation that pathogenic Mtb bacteria have evolved mechanisms to suppress IL-12 and TNF-α production [50]–[53]. On the other hand, virulent clinical strains of Mtb are found to be proficient at stimulating high levels of IL-10 and inducing immunosuppression in the host [54], [55]. Interestingly, IL-10 transgenic mice re-activate latent tuberculosis infection and overproduction of IL-10 causes increase in susceptibility to mycobacterial infection [56]. Also, in patients with active TB infection, the anti-PPD Th1 T cell responses are found to be downregulated [57], [58]. All these findings indicate an important role of the IL-12/TNF-α and IL-10 balance in the regulation of Mtb infection and disease progression; however, the mechanisms by which the bacilli influence these signaling pathways to favor their long-term survival and persistence inside the host are not well understood.

Previous work by us reveals that PPE18 is a TLR2 ligand and its binding to TLR2 results in activation of p38 MAPK, which leads to secretion of higher levels of IL-10 [22]. At the same time, this interaction also inhibits LPS-mediated IL-12 p40 and TNF-α induction involving the p38 MAPK-SOCS3-NF-κB/c-rel signaling pathway [5]. Thus, it appears that PPE18 probably plays a key role in regulating the Th1/Th2 cytokine balance, which in turn can influence bacterial persistence and multiplication inside the host [59], [60]. We have also documented earlier that the anti-PPD T cell response is skewed towards the Th2-type by PPE18 in T cells isolated from PBMCs from the BCG-vaccinated individuals, and an important role of IL-10 in the downregulation of anti-PPD Th1 response by PPE18 [22]. Thus, how much of the PPE18-induced modulation of the immune response could directly contribute to Mtb virulence *in vivo* seemed an interesting and logical issue to be looked at. In the present study, we observed that absence of *ppe18* in Mtb resulted in a marked decrease of virulence with low bacterial counts and reduced pathology in the lung, liver and spleen when compared with the WT strain in a murine model. Also we observed absence of mortality in mice infected with *ppe18* KO strain compared to mice infected with CDC1551 wild-type strain. Thus we predict that deletion of *ppe18* impairs the ability of the bacilli to induce a favorable anti-inflammatory immune environment. Although the precise mechanism of the reduced virulence of *ppe18* knock-out strain is unclear, this could be due to the fact that in the absence of PPE18, probably a better protective immune response (especially Th1) is generated which augurs well for the host [61]–[63]. It is also possible that PPE18 targets other immune effecter molecules that lie downstream of the SOCS3-NF-κB signaling cascade that has previously been shown to be affected by binding of PPE18 to TLR2 [5] to downregulate anti-mycobacterial protective immune responses. The exact reason for low numbers of *ppe18* knock-out Mtb strain *in vivo* needs to be further investigated.

The aerosol model of Mtb infection in mice fails to give rise to the classical granulomas in the lungs that are typically observed in people suffering from tuberculosis. The infected mice usually show loose aggregates of macrophages and lymphocytes that infiltrate into the lungs upon infection [64]. Such aggregates were also observed in our studies. These clusters of immune cells were seen to appear earlier in the lungs of mice infected with the WT Mtb strain. On the other hand, we observed fewer CFUs in mice infected with the *ppe18* KO Mtb strain, that resulted in delayed onset of lung, liver and spleen pathology as compared to the WT Mtb strain. This is well reflected in the absence of mortality in mice infected with *ppe18* KO strain whereas mice infected with WT Mtb strain failed to survive beyond 60 weeks. Within the localized environment of the granulomas, the immune cells regulate bacterial multiplication and thus control the spread of infection within the lungs and to other organs [65]. Various studies have indicated that even though the bacterial replication slows down after 3–4 weeks of infection, the infected mice might eventually die as a result of organ damage caused by the bacteria [29], [66]. We observed that the number of WT bacteria after aerosol infection increased at 3 weeks from day 1 and then decreased when examined at 6 weeks and 9 weeks time point. However, in these mice, we observed exacerbated lung as well as liver and spleen pathology at later time points (21 weeks and 60 weeks) which was associated with increased mortality. The decrease in CFU counts in WT-infected mice at later time points like 6 weeks and 9 weeks compared to 3 weeks time point post infection as observed in our study could be due to induction of an effective immune response, thereby keeping the intracellular bacterial load down. However, at all the time points studied, tissue pathology was found to be much less severe in mice infected with *ppe18* KO strain explaining the absence of mortality in these animals.

Mycobacterial virulence factors are defined as the traits that cause disease progression in host. The CFU load, histopathology and survival of the host after bacterial infection determine the degree of virulence. The growth of Mtb inside a host is an important indicator of virulence. Thus, observed low CFU counts of *ppe18* KO Mtb strain in mice and longer survival of the mice infected with *ppe18* KO strain could possibly mean that PPE18 is a virulence factor of Mtb. Correlations between reduced pathology accompanied by improved survival of the host have been made earlier in studies using Mtb strains deficient in mce operons [67] and the serine/threonine kinase PknG [68]. In both these studies, multiplication of the knock-out strains was found to be compromised *in vivo*. Similarly, mice infected with ΔsigC mutant had CFU counts in the lung homogenates one log unit lower than the wild-type CDC1551 and ΔsigC complemented mutant groups at day 28 and this observation persisted up to day 120 [69]. ΔsigC mutant-infected mice show milder lung pathology and remained healthy and alive for an extended time in comparison to the CDC1551 and ΔsigC complemented mutant groups [69]. Interestingly, ΔsigC mutant strain also proliferated poorly in guinea pig models [69]. Similar results were also observed when guinea pigs and mice were infected with Mtb ΔdosR mutant strain [70] or with Mtb strain harboring several sigma factor mutants [71]. All these observations indicate that poorer bacterial growth and reduced lung pathology in animal models can contribute to prolonged host survival upon infection with mutant Mtb strains [27], [67]–[75].

The PPE family comprises of 69 acid-rich members. PPE proteins such as PPE44 (Rv2770c), Rv2608, Rv1168c, Rv2430c etc have been found to elicit potent B and T cell responses [76]–[79]. Interestingly, Rv0485, the transcriptional regulator of the *pe13/ppe18* pair has been shown to be a virulence factor in a murine model of Mtb infection [17]. Our studies, however, focus directly on the role of PPE18 in Mtb virulence. This study specially gains relevance because PPE18 is a component of Mtb72f, a Mtb subunit vaccine which is currently in clinical trials [80]. Therefore, it becomes imperative to study and understand the function of PPE18 in Mtb virulence. PPE18 is thought to be present on the bacterial surface [22], [81]. Results presented in this study highlight the important role of PPE18 in replication and survival of Mtb *in vivo*. Absence of PPE18 perhaps slows down the rate of bacterial replication *in vivo*. Therefore, antibodies and other therapeutic strategies that target the PPE18 protein might help in controlling Mtb infection in a better way.

## Supporting Information

Figure S1 Histopathology of liver of mice infected with either wild-type (WT) or ppe18 KO M. tuberculosis strain at higher magnification. H&E-stained liver sections from WT (left panel) and ppe18 KO (right panel) strain infected mice were observed at 100X magnification. Arrows indicate the sites of lymphocytic infiltration.
(TIF)

Figure S2 Effect of M. tuberculosis wild-type (WT) or ppe18 KO infection on weight of mice. Weight of mice was taken 9 weeks after they were infected with either WT or ppe18 KO strains of M. tuberculosis. The percentage increase in weight was calculated with respect to the average weight of 4–6 weeks old uninfected mice. Data shown is mean ± SEM for a total of 12 mice.
(TIF)
