# Supplementary figures and images for: Role of PPE18 Protein in Intracellular Survival and Pathogenicity of Mycobacterium tuberculosis in Mice
Source: PLoS One. 2012 Dec 28;7(12):e52601. doi: 10.1371/journal.pone.0052601 (PMC3532481; doi:10.1371/journal.pone.0052601)

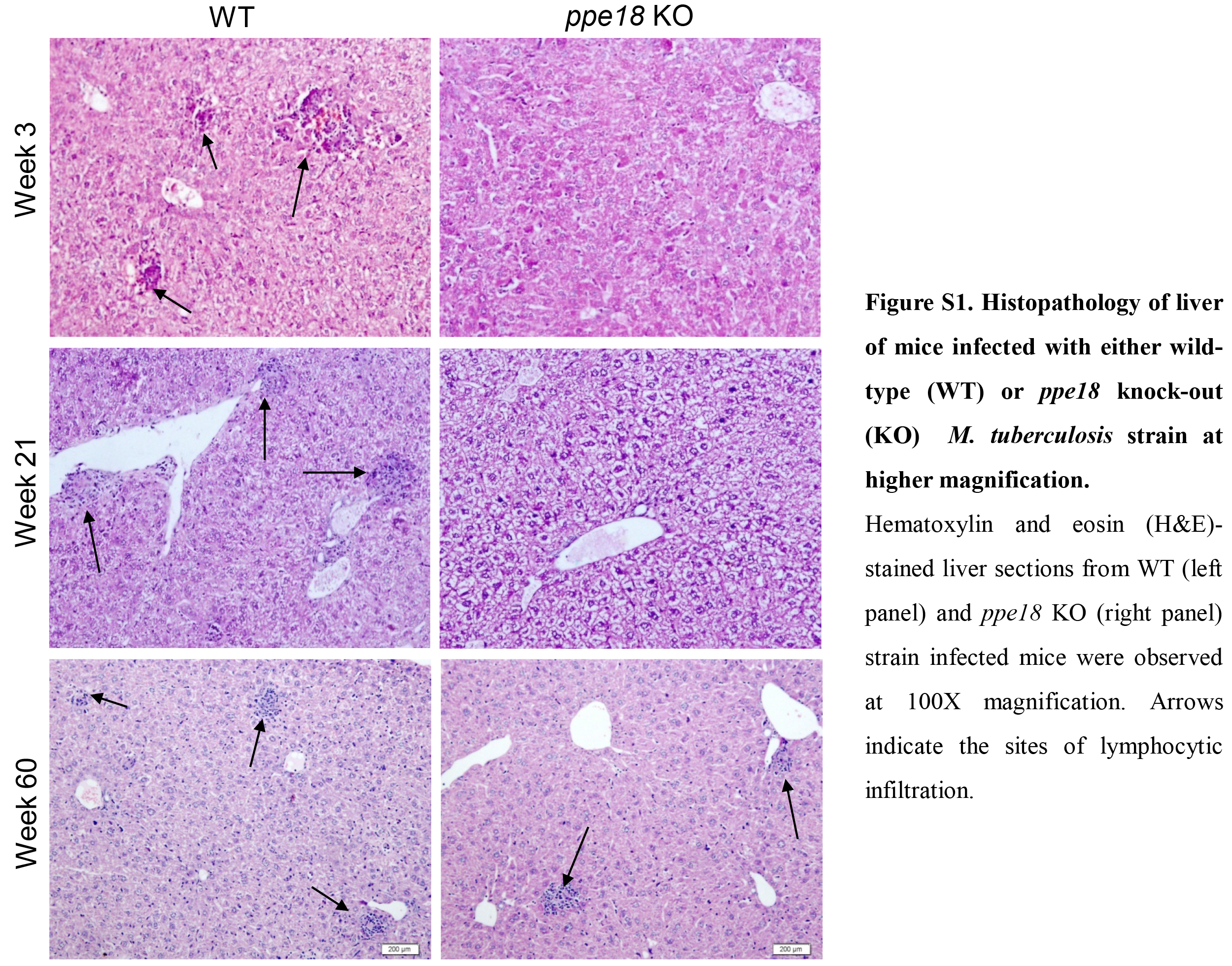

Supplement: Figure S1 — Histopathology of liver of mice infected with either wild-type (WT) or ppe18 KO M. tuberculosis strain at higher magnification. H&E-stained liver sections from WT (left panel) and ppe18 KO (right panel) strain infected mice were observed at 100X magnification. Arrows indicate the sites of lymphocytic infiltration. (TIF) [file pone.0052601.s001.tif]

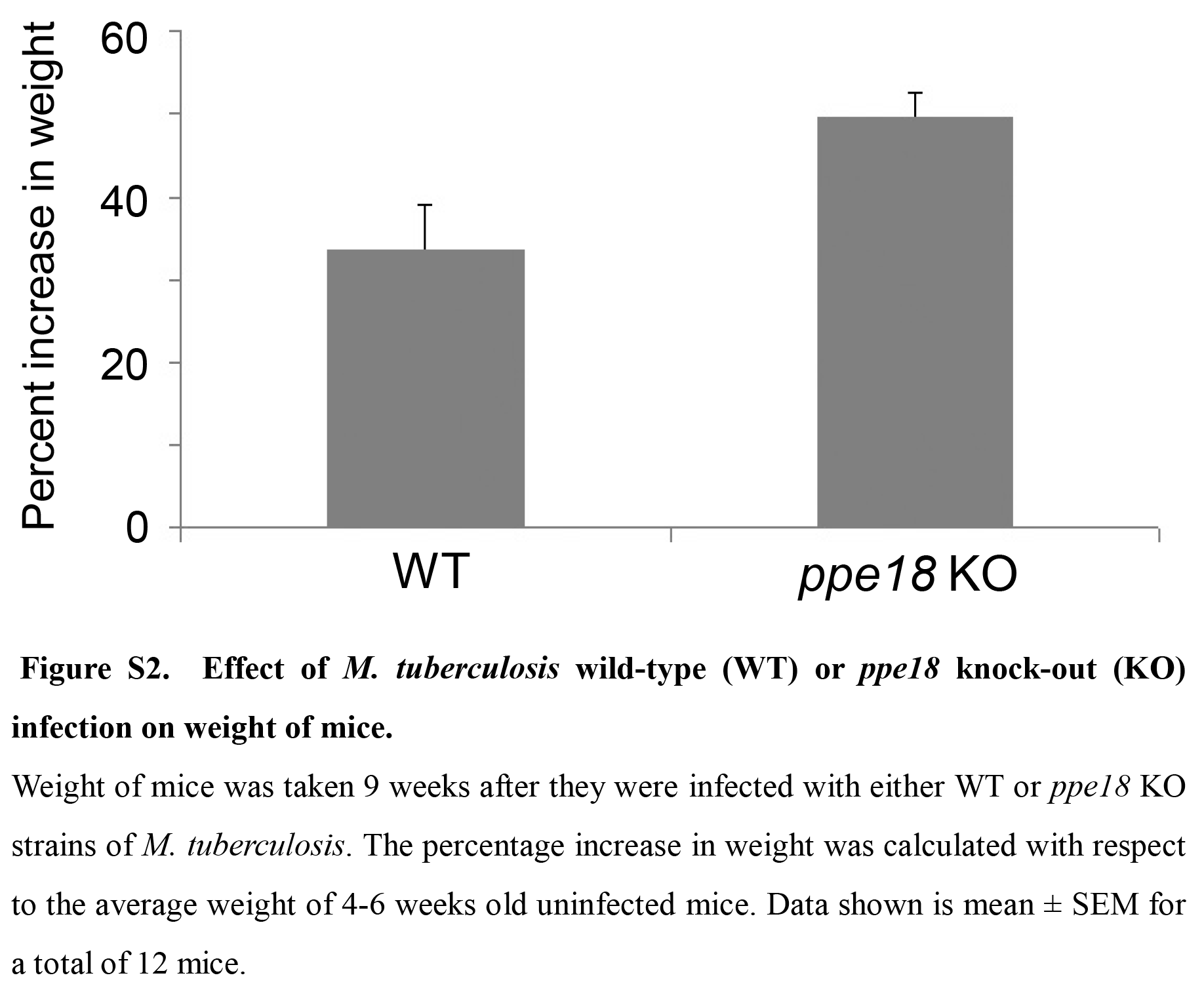

Supplement: Figure S2 — Effect of M. tuberculosis wild-type (WT) or ppe18 KO infection on weight of mice. Weight of mice was taken 9 weeks after they were infected with either WT or ppe18 KO strains of M. tuberculosis. The percentage increase in weight was calculated with respect to the average weight of 4–6 weeks old uninfected mice. Data shown is mean ± SEM for a total of 12 mice. (TIF) [file pone.0052601.s002.tif]
